# The Micro-Flow Mechanism of Polymer Flooding in Dual Heterogeneous Reservoirs Considering the Wettability

**DOI:** 10.3390/polym15204188

**Published:** 2023-10-23

**Authors:** Huiying Zhong, Bowen Shi, Yuanyuan He, Yongbin Bi, Yu Zhao, Kun Xie

**Affiliations:** 1 Key Laboratory for Enhanced Oil & Gas Recovery of the Ministry of Education, Northeast Petroleum University, Daqing 163318, China; 15946294624@163.com (B.S.); byb5800@163.com (Y.B.); xiekun725@163.com (K.X.); 2 Jianghan Oilfield Company, SINOPEC, Qianjiang 430070, China; rainbow_hyy@126.com; 3 Nanpu Operation Area, PetroChina Jidong Oilfield Company, Tangshan 063200, China; 4 Exploration & Development Research Institute, PetroChina Daqing Oilfield Company Limited, Daqing 163712, China; zhaoyu6868@petrochina.com.cn

**Keywords:** wettability, heterogeneous reservoir, polymer flooding, micro-flow mechanism, oil displacement efficiency

## Abstract

There have been some studies conducted about the single factor viscoelasticity of polymer solution or wettability effect on the micro-flow mechanism of polymer flooding. In this paper, the flow mechanism of polymer solution in dual heterogeneous reservoir considering the wettability and gravity was studied. The influences of wettability and rock particle shape on flow characteristics were studied based on the characteristics of saturation and pressure distribution. Compared with the simulation results of polymer flooding in three different rock particle shapes porous media, the oil displacement efficiency of the circular particle model is the highest at 91.57%, which is 3.34% and 11.48% higher than that in the hexagonal and diamond models, respectively. The influence of wettability was studied by the circular particle model. The oil displacement efficiency under water-wet conditions was higher than that under oil-wet conditions. The displacement process considering gravity was affected by the crossflow caused by gravity and viscous force, and the micro-oil displacement efficiency was 9.87% lower than that of non-gravity. Considering the wettability, vertical crossflow will be formed. The oil displacement efficiency under water-wet conditions was 3.9% higher than in oil-wet conditions. The research results can not only expand and enrich the micro-flow mechanism of viscoelastic polymer solution, but also provide reference and guidance for polymer flooding scheme design.

## 1. Introduction

**Figure 1:**
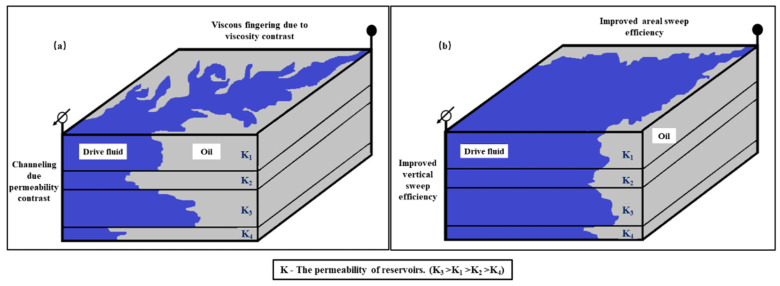
Schematic of volumetric sweep improvement achieved by polymer flooding [11,12]: (**a**) water flooding; (**b**) polymer flooding.

Polymer flooding has come into the commercial applications stage in oilfields, including use in viscous oil reservoirs, conventional oil reservoirs, and offshore oil reservoirs [1,2]. Polymer flooding can significantly improve oil recovery by enhancing the oil-water flow ratio and increasing the sweep coefficient [3,4,5], as shown in Figure 1. Figure 1 illustrates the improvement in volumetric sweep efficiency in a heterogeneous reservoir (*K*_3_ > *K*_1_ > *K*_2_ > *K*_4_) by maintaining conformance and mobility control through polymer flooding. The *K* represents the permeability of reservoirs. However, polymer flooding is still facing problems such as a sharp increase in water cuts [6], deterioration of the tertiary oil recovery environment [7], and difficulty in displacing the dead-end or dead oil [8], which brings challenges to improving the crude oil recovery of polymer flooding in oilfields [9,10].

In recent years, the study on the micro-flow mechanism of polymer flooding has been mainly concentrated in the laboratory [13,14], and these studies focus on the effect of polymer solution viscoelasticity on the oil displacement efficiency and micro-flow mechanism [15,16]. Wang et al. concluded that the mechanism of polymer solution in improving microscopic oil displacement efficiency is elasticity by core displacement experiments [17]. Xia et al. employed microscopic flow experiments to demonstrate that the larger the viscoelasticity of the polymer solution is, the more residual oil is displaced, and the higher the oil displacement efficiency is [18]. Clemens et al. revealed that injecting polymers could slow down the finger behavior compared to water flooding by numerical simulations, and proved that the polymer displacement efficiency was affected by the shear thinning behavior [19]. Through computed tomography (CT) scanning technology, Tan et al. found that polymer solution to displace the residual oil after water flooding is mainly achieved by the shear and tugging effect of the polymer solution [20].

It is generally believed that the interlayer heterogeneity of reservoirs can also affect oil displacement efficiency and the micro-flow mechanism for polymer solution [21]. Fang et al. conducted a study on polymer flooding in a photolithography glass model. They observed that with the increase in polymer molecular weight, the oil displacement efficiency of the high-permeability model increased, and that of the low-permeability model first increased and then decreased [22]. Xin et al. demonstrated that the critical pressure gradient of heavy oil is higher in the low-permeability porous media than in the high-permeability porous media during polymer flooding in heterogeneous heavy oil reservoirs using polymer flooding experiments and simulations [23]. Zhong et al. developed a solver based on the OpenFOAM platform considering the interlayer heterogeneity; the simulation indicated that the reservoir structure has more severe heterogeneity and worse connectivity, its oil displacement efficiency is relatively low, and the commingled production has a stronger effect on the micro-oil displacement efficiency of a poorer pore structure layer than that of a better pore structure layer [24]. Zhang et al. carried out polymer flooding experiments using different core permeability and porosity cores; they observed that polymer flooding is mainly effective for pores larger than 40 μm by comparing the oil saturation before and after polymer flooding using nuclear magnetic resonance spectroscopy technology [25]. Meanwhile, other studies have considered the effect of gravity on the flow mechanism and the oil displacement efficiency of viscoelastic polymer solution [26]. Fu et al. classified three types of vertical remaining oil, including the interlayer occlusion type, rhythm type, and gravity type. The proportion of the gravity type was more than 30% [27]. Hamidpour et al. investigated the effect of gravity, viscous force, and capillary force on polymer flooding, and found that viscous force plays a dominant role, and gravity and capillary force affect the residual oil distribution [28]. Rezaeiakmal et al. investigated the effect of gravity on polymer flooding in homogeneous pores under the effect of bubbles by microscopic visualization experiments to determine that the recovery of residual oil can be substantially improved [29].

Therefore, this paper further studies the micro-flow mechanism of a viscoelastic polymer solution under the conditions of dual heterogeneous reservoirs, considering wettability and gravity, on the basis of research on the viscoelastic polymer flooding mechanism and the application effect of the Daqing Oilfield (China). This study involves the effects of rock particles, wettability, and gravity, which makes it more suitable for the actual flow of polymer solution in porous media, and could also be a supplement for the limitations of experimental studies. The microscopic double layer heterogeneous pore model of the different rock particles is constructed. The relationship between rock particle shape on microscopic oil displacement efficiency is characterized. The effects of different wettability conditions and the degree of wettability on the microscopic oil displacement efficiency are discussed and validated by numerical simulations. Furthermore, the formation process and conditions of columnar residual oil are described by numerical simulation results. The work is helpful to promote the development of the micro-flow mechanism of polymer flooding, and can also provide a scientific basis for the polymer flooding of heterogeneous reservoirs to improve oil recovery.

## 2. Modeling

### 2.1. Mathematical Model

In this paper, the oil-water two-phase flow is studied, and the continuity equation and Navier–Stokes dimensionless equations are written as [30,31]

(1) ∇⋅U=0

(2) ∂U∂t+U⋅∇U=1Re−∇p+∇2U+1Fr2g+1Weκ∇α

where ***U*** is the dimensionless velocity vector; *p* is the dimensionless pressure; *g* is the dimensionless gravitational acceleration; *Re* is the Reynolds number and it is defined as $\frac{\rho VL}{\mu }$; *Fr* is the Froude number and it is defined as $\frac{V}{\sqrt{gL}}$; *α* is the phase fraction of two phases and it takes values between 0 and 1, 1 for polymer solutions and 0 for oils; *We* is the Weber number and it is $\frac{\rho L^{2}}{\sigma }\cdot \frac{V^{2}}{L}$; *V* is the characteristic velocity, and it takes the average velocity of inlet, m/s; *L* is the characteristic length and it usually takes the inlet length of the model, m; *σ* is the interfacial tension between the oil and the polymer solutions, N/m; *ρ* is the density of fluid, kg/m^3^; *μ* is the dynamic viscosity of fluid, Pa·s.

The phase equation and fluid properties are given by [32,33]

(3) $$\frac{\partial \alpha }{\partial t}+\nabla \cdot \left(U\alpha \right)=0$$

(4) $$\rho =\alpha \rho_{1}+\left(1-\alpha \right)\rho_{2}$$

(5) $$\mu =\alpha \mu_{1}+\left(1-\alpha \right)\mu_{2}$$

where *ρ*_1_ and *ρ*_2_ represent the density of displacement fluid and displaced fluid, respectively, kg/m^3^; *μ*_1_ and *μ*_2_ represent the dynamic viscosity of displacement fluid and displaced fluid, respectively, Pa·s.

In Equation (2), ***κ*** is the mean curvature of free surface and its expression is shown as [34,35,36]

(6) κ=−∇⋅n

where *n* is the interface normal vector, which can be expressed by

(7) $$n=\frac{\nabla \alpha }{\left|\nabla \alpha \right|}$$

During polymer flooding, the capillary number (*Ca*) is often employed to reflect the balanced relationship between different forces. It is a dimensionless quantity which was originally proposed by Taylor in 1934. It is defined as the ratio of the viscous force to the capillary force, given by

(8) $$Ca=\frac{\mu \cdot V}{\sigma }$$

### 2.2. Boundary Conditions

In this paper, the influence of wettability on the flow characteristics of the solid is considered. The normal vector of the bulk surface is expressed as [37,38]

(9) $$n=n_{w}\cos\theta +t_{w}\sin\theta$$

where *n*_w_ is the wall unit normal vector; *t*_w_ is the wall unit tangent vector; *θ* is the contact angle.

Wettability is commonly characterized by the contact angle between the grain surface, oil, and water. Because wettability depends upon mineralogy, oil composition, formation water chemistry, and reservoir temperature, the contact angle can take on any value from 0° (water wet) to 90° (neutral wet) and from 90° to 180° (oil wet). The contact angle is the angle between the solid surface plane and the tangent to the oil drop surface plotted at the point of contact between three phases (oil, water, and solid).

The inlet is defined as a velocity inlet and the outlet as a pressure outlet, with a reference pressure of 0 and backflow inhibition. The fluid is incompressible and laminar flow in the micro-models. The two-phase flow of polymer and crude oil in the porous media at micro-scale is simulated based on the above equations and boundary conditions.

The equations are solved based on the PISO algorithm and the procedure is as follows:

1. Give initial value *α*, *V*, ***U***, *p*, *ρ*, *μ,* obtain parameters *Re*, *We*, *Fr*, *Ca*;
2. Solve the Equation (3) to obtain an updated volume fraction *α*′;
3. Solve Equation (2) to get an intermediate velocity value ***U***′;
4. PISO-Loop, and obtain ***U***″;
5. Increase the time step, go back to the first step, and loop steps (1)~(4) until the accuracy requirement is met.

### 2.3. Physical Model

**Figure 2:**
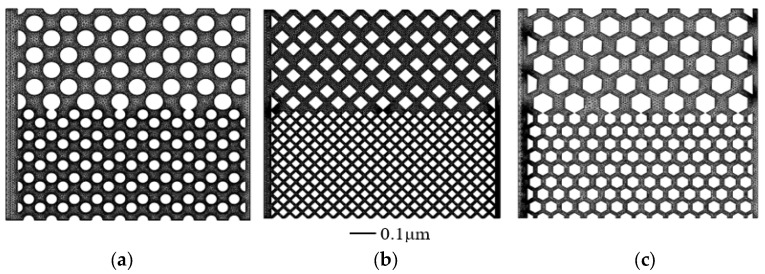
Dual heterogeneous model for three different rock particle shapes: (**a**) circular particle; (**b**) diamond particle; (**c**) hexagonal particle.

Three different particle shapes were used to represent the different microscopic rock characteristics. The pattern of particles arrangement and the topology is the same, therefore the micro-flow mechanism was not affected by other factors. The property of the dual heterogeneous model is shown in Figure 2, which is constructed to study the effect of heterogeneous properties with the same particle shape and topology.

The structure of the solid was classified into three types according to the scanning results of the sampled cores based on different roundness and sorting properties. In order to reflect the influence of the solid particles’ shape on the polymer flooding process more clearly, the circular particles were taken to represent the particles with the best roundness and sorting. The hexagonal particles and diamond particles were employed to represent the particles with the worst roundness and sorting properties.

**Figure 3:**
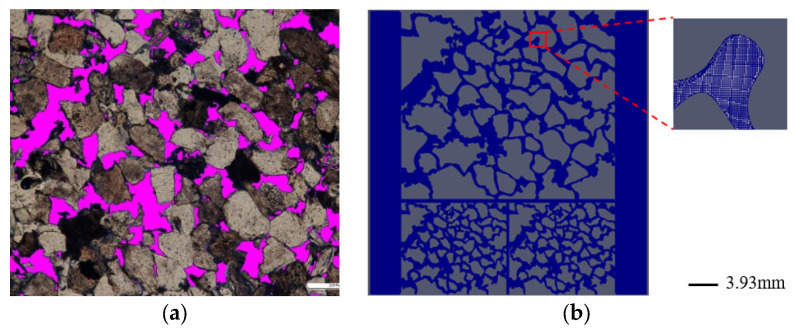
Model of a complex dual heterogeneous pore: (**a**) CT scan image; (**b**) mesh profile of a two-layer heterogeneous model.

Microscopic observation of the cast thin section of the core can truly reflect the size and connectivity of the pore and throat. In order to simulate the flow process and state of residual oil after polymer flooding, a complex pore model was constructed on the basis of the core cast thin section. Thus, in this paper, the complex pore model basis on the cast thin section of the core from Daqing Oilfield (China) was established [39], as shown in Figure 3a. Commingle production is usually used in actual oilfield development. The heterogeneity between vertical layers can affect the vertical sweep efficiency, but the micro mechanism of the vertical heterogeneity effect on the displacement efficiency is not very clear. Thus, in this study, the single layer model is scaled down to a low-permeability model. Then, these two models are stitched together to form a dual heterogeneous model, which could ensure the same connectivity of two layers. So, the simulation results are only affected by heterogeneous characteristics. Meanwhile the model could also simulate the effect of polymer flooding considering gravity [35], and its schematic of geometries and mesh arrangements are shown in Figure 3b.

## 3. Results and Discussion

### 3.1. Effect of Particle Shape on Micro-Flow Characteristics

#### 3.1.1. Oil Saturation Distribution

**Figure 4:**
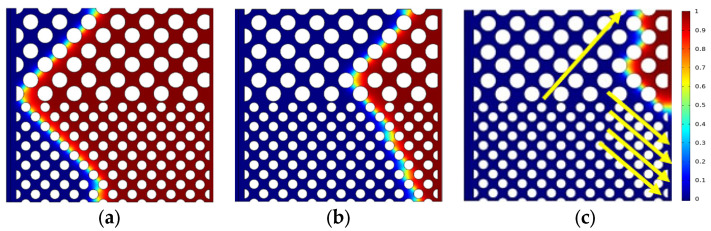
Distribution of oil saturation in the circular particle model: (**a**) *t* = 0.174; (**b**) *t* = 0.464; (**c**) *t* = 0.638.

**Figure 5:**
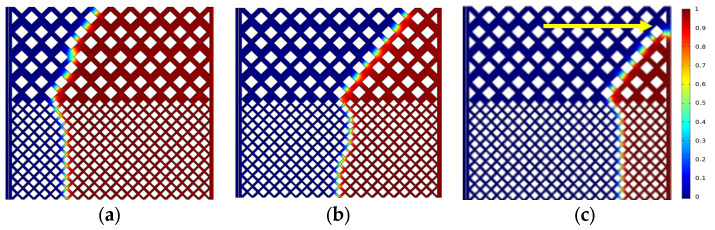
Distribution of oil saturation in the diamond particle model: (**a**) *t* = 0.174; (**b**) *t* = 0.464; (**c**) *t* = 0.638.

**Figure 6:**
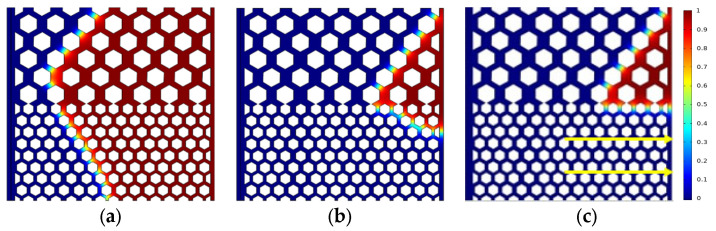
Distribution of oil saturation in the hexagonal particle model (*Fr* = 0.00319, *We* = 2.7 × 10^−12^): (**a**) *t* = 0.174; (**b**) *t* = 0.464; (**c**) *t* = 0.638.

The oil saturation distribution for different displacement processes is shown in Figure 4, Figure 5 and Figure 6 with the same capillary number (*Ca* = 2.4 × 10^−5^) for different particle shapes. Under reservoir conditions, the flow is usually in a situation dominated by capillary force (low *Ca*). The solid wall is neutral wet in this simulation. The color legend of the simulation results represents the oil saturation, and the simulation results are shown in red for crude oil and blue for polymer solution. Meanwhile, the *t* represents the dimensionless displacement time. The *Fr* and *We* represent the Froude number and Weber number, respectively. The *Fr* is the ratio of inertial force to gravity, and reflects the stability of the flow. When the *Fr* is less than 1, the fluid flow is stable, and gravity plays a dominant role. The *We* is generally employed to denote the relative importance of the relationship between the inertial forces and interfacial tension of fluid flow.

Figure 4 indicates that the upper part of the high-permeability layer and the lower part of the low-permeability layer have a relatively high flow rate, whereas the central region has a relatively slow flow rate due to the heterogeneity of the interlayer, which interferes with the flow. As shown in Figure 4c, the flow direction of displacement front is 45° from the horizontal line (e.g., yellow solid arrows) due to the main flow channel direction. There are only four channels in the high-permeability layer connecting with the outlet, while there are seven channels in the low-permeability layer, crude oil is displaced more easily in the low-permeability layer (multi-channel region). That is why there is residual oil in the high-permeability layer. The main flow channel direction of the diamond-shape particle model is 45° to the outlet in the high-permeability layer, as shown in Figure 5c. Although the diamond particle model has more flow channels connecting with the outlet in the low-permeability layer, its worst sorting and small pore throat radius result in residual oil retention in the low-permeability layer at a dimensionless time of 0.638. Similarly, for the hexagonal particle model, the displacement front in the high-permeability layer is 45° to the outlet as the same as the circular particles, as shown in Figure 6c, while the initial displacement in the low-permeability layer is 45° (at dimensionless time of 0.464), and with the increase in the displacement time, the main flow path is vertical to the outlet due to its better roundness and sorting than that of the diamond particles, resulting in the eventual formation of residual oil in the high-permeability layer.

**Figure 7:**
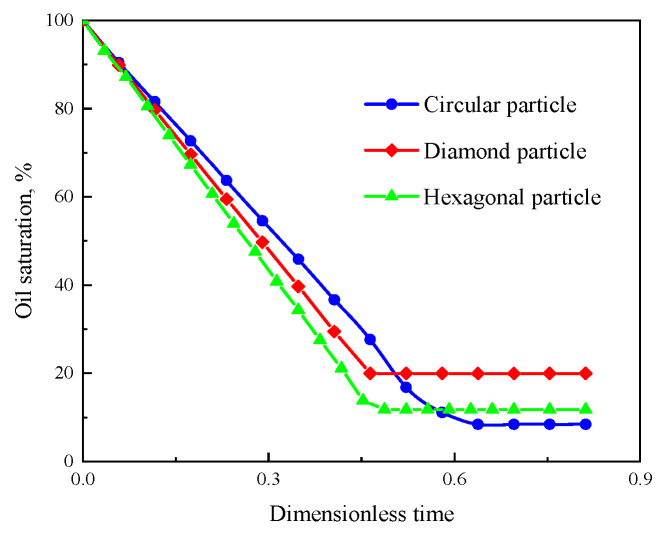
Oil saturation variation of different rock particles with times.

Figure 7 shows the oil saturation during the displacement process in the three models. The oil saturation in the diamond particle model is the first to reach an equilibrium state, without further reduction when the displacement front of the polymer solution breaks through. The displacement front breakthrough in the circular and hexagonal model is at 0.58 and 0.452 (the dimensionless displacement time), respectively, and then the oil saturation curve decreases at a slower rate and gradually reaches an equilibrium state. As shown in Figure 7, the displacement of three solid particle shapes reaches an equilibrium state when the dimensionless time is 0.8, the oil saturation of the circular particles, diamond particles, and hexagonal particles are 8.43%, 19.91%, and 11.77%, respectively. Therefore, it could be concluded that the oil displacement efficiency is 91.57%, 80.09%, and 88.23%, respectively. The oil displacement efficiency of the circular particle model is the highest with 91.57% due to the high sorting property and low flow resistance, which is 3.34% and 11.48% higher than that in the hexagonal and diamond particle models, respectively. The results of the oil saturation distribution show that the better the sorting properties of the particles, the lower the resistance to flow, and the higher the oil displacement efficiency.

#### 3.1.2. Pressure Distribution

**Figure 8:**
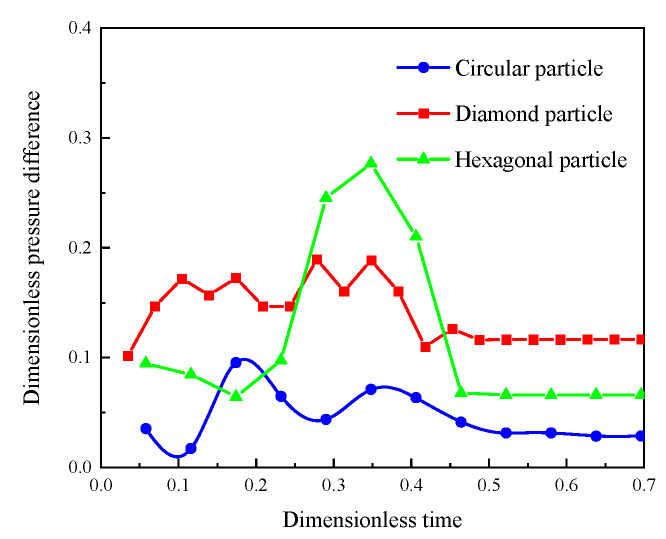
Pressure difference between inlet and outlet vs. dimensionless time for different rock particles.

**Figure 9:**
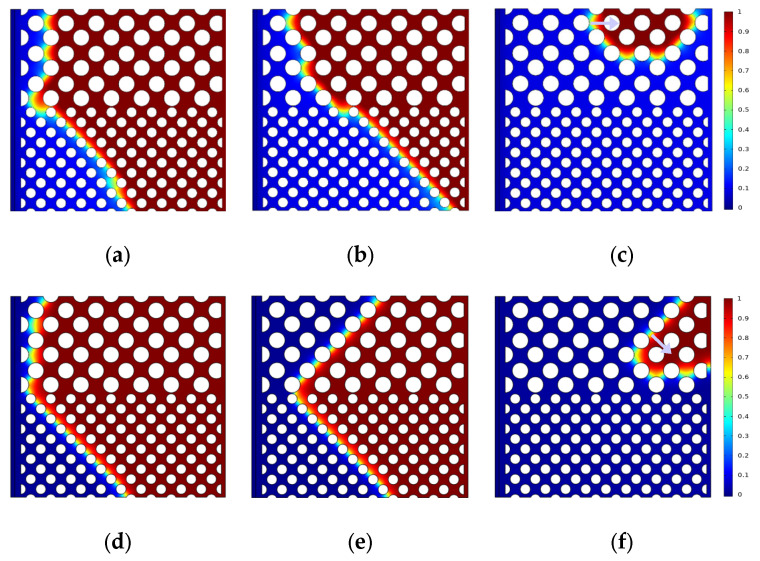
Distribution of oil saturation at different contact angles under water-wet conditions (*Fr* = 0.00319, *We* = 2.7 × 10^−12^): (**a**) *θ* = 60° *t* = 0.1; (**b**) *θ* = 60° *t* = 0.232; (**c**) *θ* = 60° *t* = 0.812; (**d**) *θ* = 80° *t* = 0.116; (**e**) *θ* = 80° *t* = 0.232; (**f**) *θ* = 80° *t* = 0.464.

Figure 8 shows the pressure difference between the inlet and outlet in three different models. In the initial stages of displacement, there is a certain initial pressure, and the worse the roundness of the particle shape, the higher the initial pressure. At the middle of the displacement, the polymer solution starts to displace the oil in the lower permeability layers with slight fluctuations in the displacement pressure difference, where the worst storability of the diamond particle model causes the greatest fluctuations in the displacement pressure difference due to the greatest resistance. The sudden increase in the displacement pressure difference for the hexagonal particle shape model in Figure 9 is due to the boundary between two layers, which results in an extra displacement resistance, leading to a sudden increase in the displacement pressure difference. By the end of the displacement, when the polymer displacement front breaks through, the displacement pressure difference shows a stable trend.

### 3.2. Effect of Wettability on Micro-Flow Characteristics

#### 3.2.1. Effect of Wettability on Displacement Characteristics

**Figure 10:**
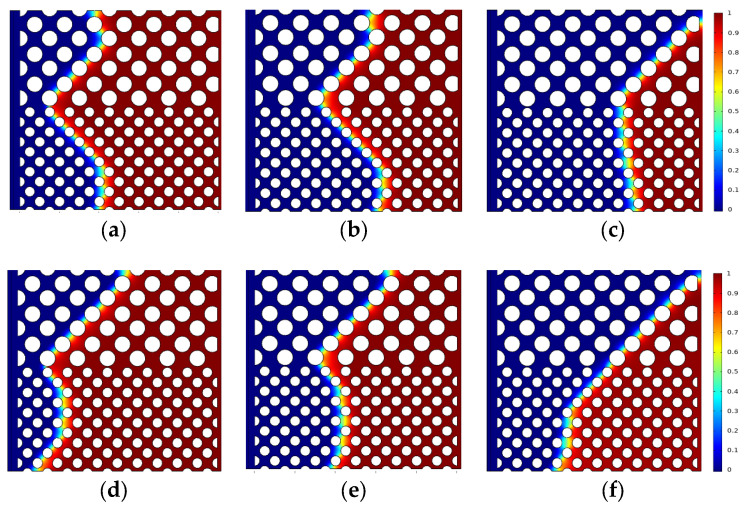
Distribution of oil saturation at different contact angles under oil-wet conditions (*Fr* = 0.00319, *We* = 2.7 × 10^−12^): (**a**) *θ* = 95° *t* = 0.232; (**b**) *θ* = 95° *t* = 0.348; (**c**) *θ* = 95° *t* = 0.638; (**d**) *θ* = 105° *t* = 0.232; (**e**) *θ* = 105° *t* = 0.348; (**f**) *θ* = 105° *t* = 0.58.

Figure 9 shows the oil saturation for water-wet conditions when capillary number is 2.4 × 10^−5^ and contact angles *θ* are 60° and 80°, respectively. Figure 10 shows the oil saturation for oil-wet conditions when capillary number is 2.4 × 10^−5^ and contact angles are 95° and 105°, respectively. The color legend of the simulation results represents the oil saturation, and the simulation results are shown in red for crude oil and blue for polymer solution. Meanwhile, the *t* represents the dimensionless displacement time. The *Fr* and *We* represent the Froude number and Weber number, respectively. The *Fr* is the ratio of inertial force to gravity, and reflects the stability of the flow. When the *Fr* is less than 1, the fluid flow is stable, and gravity plays a dominant role. The *We* is generally employed to denote the relative importance of the relationship between inertial forces and interfacial tension of fluid flow.

When the solid wall is water-wet, the capillary force could be regarded as the driving force, and the polymer solution is preferred for the displacing crude oil in the low-permeability layer, as shown in Figure 9. The injected polymer solution will enter the tiny pores under the capillary force, which can effectively improve the micro-oil displacement efficiency of the low-permeability layer. As shown in Figure 9, it is evident that the displacement front direction is the same as that in Figure 5 from the simulation results. Under water-wet conditions, the smaller the contact angle (60° < 80°, strong water-wet conditions) is, the stronger the capillary force in the low-permeability layer is. This results in the oil of the low-permeability layer under 60° contact angle conditions being completely displaced in the early moment. Compared to Figure 9c,e, the direction of the capillary force is vertical to the solid particle under the 60° condition, which results in the capillary force not playing the role of displacing oil to the outlet (e.g., the light blue arrow in Figure 9c). In 80° contact angle conditions, the direction of the capillary force is along the main flow path (as shown by the light blue arrow in Figure 9e), which is also the main reason for the different distribution of residual oil under 60° and 80° contact angle, and higher oil displacement efficiency. For the oil-wet conditions, the capillary force is the displacement resistance, and there are more large pores and less viscous resistance in the high-permeability layer; therefore, the polymer solution preferentially displaces the crude oil from the high-permeability layers, as shown in Figure 10. This indicates that oil displacement efficiency under water-wet condition is much higher than that under oil-wet conditions during the same displacement time.

**Figure 11:**
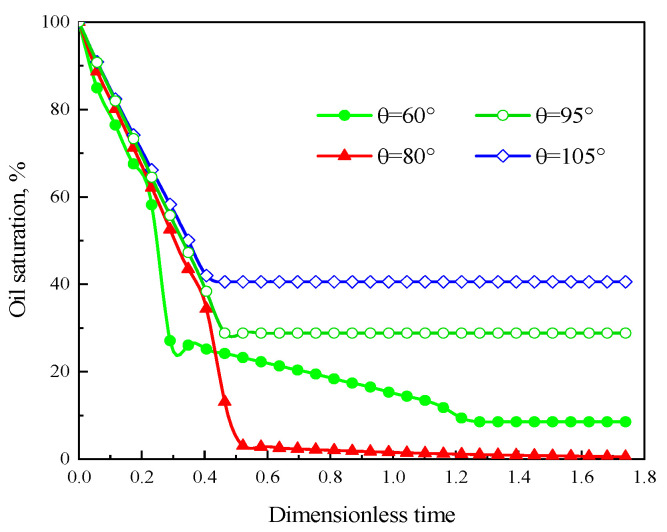
Oil saturation variation curve with time under different contact angle conditions.

**Figure 12:**
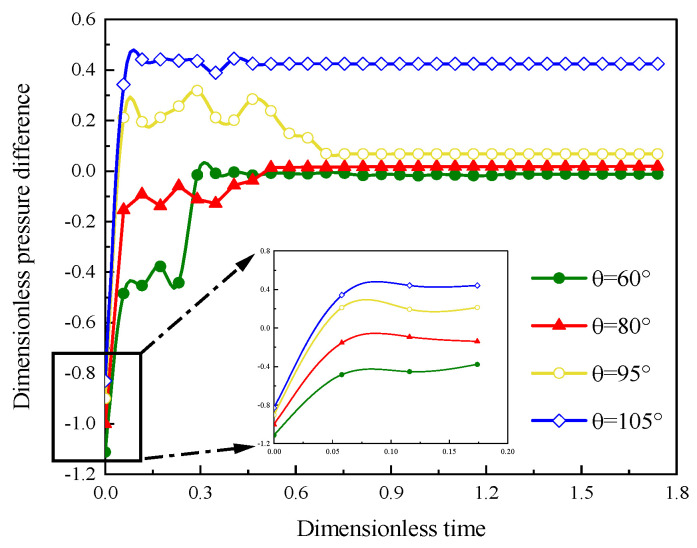
Displacement pressure differences vs. dimensionless time with different contact angles (the inset figure shows a partial large-scale view in the early displacement phase).

Figure 11 is the oil saturation vs. dimensionless time with different contact angles. It can describe the oil saturation decrease under different contact angle conditions quantitatively. At the beginning of displacement, the smaller the contact angle, the faster the oil saturation decreases. With the increase in the injection time, the slope of the curve is significantly different with different contact angle. Combined with Figure 9 and Figure 10, under water-wet conditions, the oil in the higher permeability layers is displaced more preferentially; the main flow path is formed with the displacement front breakthrough, then no more oil is displaced from the lower permeability layers, and the curve stabilizes after the turning point. In contrast, under water-wet conditions, the oil in the lower permeability layers is displaced firstly, then the polymer solution will also displace oil from the higher permeability layers after breakthrough, so the residual oil saturation is significantly lower than that of the oil-wet conditions. As shown in Figure 12, it is observed that the solid wall is closer to neutral wettability, the higher oil displacement efficiency. And the oil displacement efficiency under the water-wet conditions is higher than that under the oil-wet conditions. The main reason is when the solid wall is under water-wet conditions, the polymer solution can continually displace oil from the high-permeability layer after front breakthrough due to the large pores and low resistance in the high-permeability layers. However, when the solid wall is under oil-wet conditions, the small pores and high resistance of the low-permeability layer will result in a large area of oil trapped in the low-permeability layer. In conclusion, the oil displacement efficiency under water-wet conditions is greater than under oil-wet conditions, and the contact angle is closer to 90°, the higher the oil displacement efficiency.

#### 3.2.2. Characteristics of Pressure Distribution

**Figure 13:**
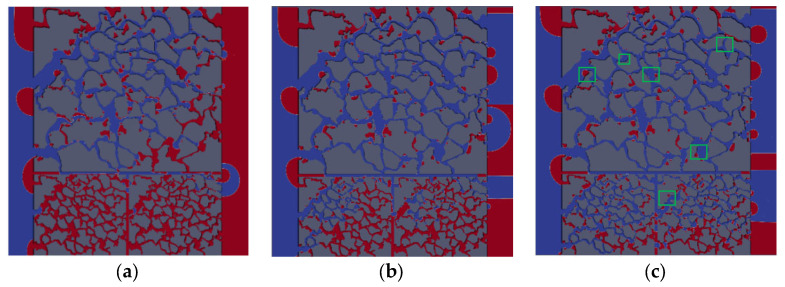
Distribution of oil saturation without gravity: (**a**) *t* = 0.18; (**b**) *t* = 0.35; (**c**) *t* = 0.68.

Figure 12 is the pressure difference between the inlet and outlet with different contact angle; the characteristic in the early stage is revealed in the local enlargement image. We can observe that the smaller the contact angle is, the smaller the initial displacement pressure difference is. With increasing displacement time, the polymer solution begins to displace the oil within the dual heterogeneous model, alternately entering the pore and throat, resulting in displacement pressure difference fluctuation, and gradually reaching a stable state. However, under water-wet conditions, the stable pressure difference is around 0, while under oil-wet conditions, the stable pressure difference is greater than 0. In Figure 13, the resistance by capillary forces leads to a higher displacement pressure difference in the oil-wet conditions than in the water-wet conditions. This indicates that the larger the contact angle is, the larger the stable pressure difference is, and also reflects the high displacement resistance and low polymer flooding displacement efficiency under oil-wet conditions.

### 3.3. Effect of Gravity on Micro-Flow Characteristics

#### 3.3.1. Effect of Gravity on Micro-Flow Mechanism of Polymer Flooding

**Figure 14:**
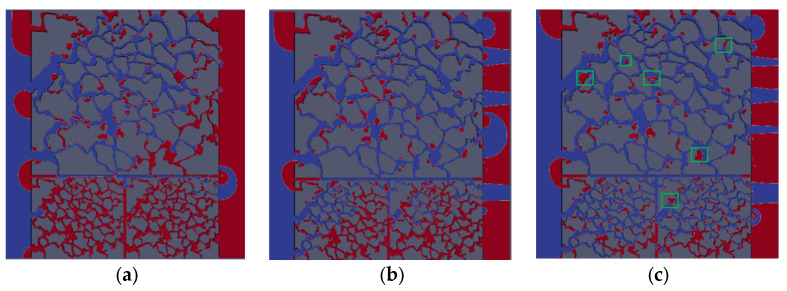
Distribution of oil saturation considering gravity: (**a**) *t* = 0.18; (**b**) *t* = 0.35; (**c**) *t* = 0.68.

In vertical dual heterogeneous reservoirs, the fluid gravity (vertically downward) will cause crossflow between the high-permeability and low-permeability layers. As shown in Figure 13 and Figure 14, when the dimensionless displacement time is 0.68, compared with no gravity, there is more oil that is difficult to be displaced considering gravity, and more columnar and clustered residual oil is formed. Thus, there is a higher oil displacement efficiency when gravity is not considered. This shows that with the increase in displacement time, the crude oil in the high-permeability layer will be displaced preferentially due to the influence of its own large pore, small resistance, and vertical crossflow effect, while the oil in the low-permeability layer, it is relatively more difficult to be displaced. Therefore, this leads to the formation of residual oil in the low-permeability layer after the priority breakthrough of the displacement front, which is difficult to displace.

Compared with the oil saturation distribution of no gravity (Figure 13), it is obvious that gravity plays a significant role on the cluster residual oil and columnar residual oil. This phenomenon is due to the cluster and columnar residual oil being mostly contained in the vertical connected throat in the pore channel; however, the flow is horizontal, and the gravity of vertical direction will have a significant effect on this type of residual oil [40].

The difference in oil displacement efficiency between the two cases is shown after the front breakthrough. There is a vertical pressure gradient between the layers, resulting in flow from the high-permeability layer to low-permeability layer, and more oil crossflow to the low-permeability layer caused by downward gravity [41]. The oil in the low-permeability layer is difficult to be displaced due to tiny pores and large flow resistance. Ultimately, the oil displacement efficiency considering gravity is 9.87% lower than that of no-gravity displacement.

#### 3.3.2. Effect of Wettability on Micro-Flow Mechanism of Polymer Flooding

**Figure 15:**
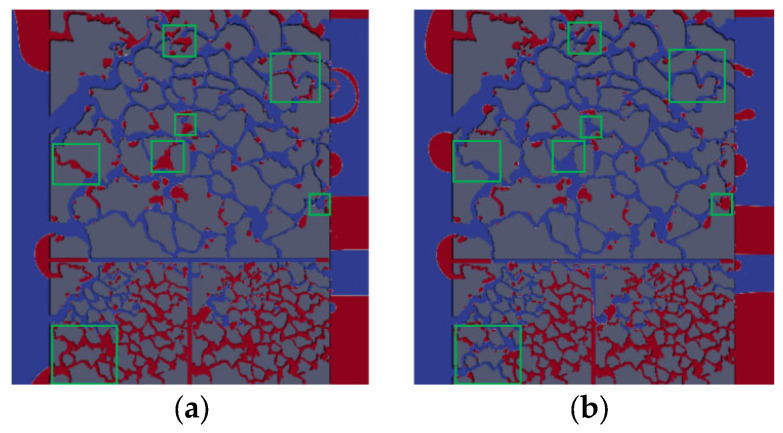
Distribution of oil saturation at the same displacement time (*t* = 0.34): (**a**) *θ*_0_ = 120°; (**b**) *θ*_0_ = 60°.

The wettability has a significant influence on the distribution of the residual oil in the porous medium, and determines the magnitude and direction of the capillary forces. Hence, the effect of wettability on the micro-flow mechanism and residual oil distribution is investigated based on a dual heterogeneous model considering gravity. Figure 14 shows the distribution of oil saturation under oil-wet and water-wet conditions at the same displacement time. The oil distribution in the area circled by a green solid line is different between the right and the left. As shown in Figure 15, the oil saturation on the left is lower than that on the right, so the oil displacement efficiency considering gravity is lower than that without gravity. Therefore, the local displacement efficiency under oil-wet conditions is lower than that under water-wet conditions. Analysis of these areas shows that the capillary force under water-wet conditions is the driving force, and has a significant displacement effect on columnar and drop residual oil, with a higher oil displacement efficiency, even higher than 3.9% of the oil displacement efficiency under oil-wet conditions.

**Figure 16:**
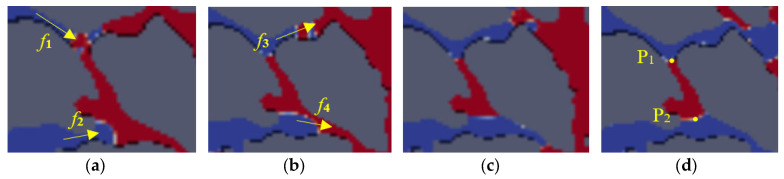
The formation process of columnar residual oil: (**a**) *t* = 0.108; (**b**) *t* = 0.11; (**c**) *t* = 0.113; (**d**) *t* = 0.119.

**Figure 17:**
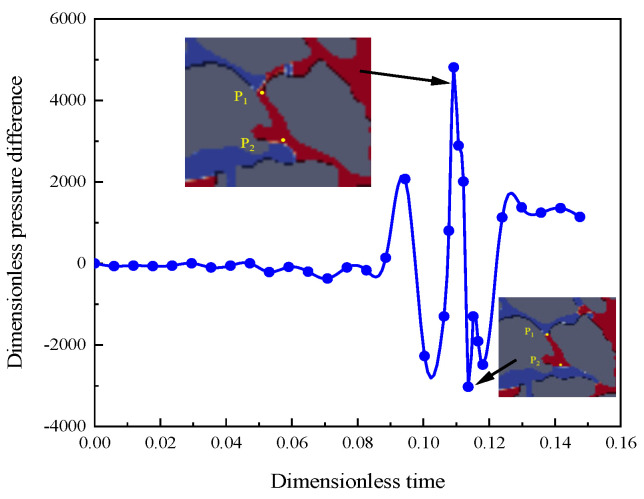
Dimensionless pressure difference between points P_1_ and P_2_ with time.

How columnar residual oil is formed is revealed when gravity is considered in the displacement process. The local amplified image is as shown in Figure 16. Firstly, the specific pore structure is a necessary condition for the formation of columnar residual oil [42], as shown in Figure 16a. In the early displacement stage, the displacement phase flows preferentially along the flow paths in the *f*_1_ and *f*_2_ directions to overcome the capillary resistance into the pore which displaces the crude oil phase. Then, the polymer solution bypasses two ends of the column of residual oil, which are the points P_1_ and P_2_ in Figure 16d. However, the displacement phase does not displace the crude oil in the columnar pore space; it flows in the direction of the main flow paths, which are *f*_3_ and *f*_4_ in Figure 16b. Eventually, the residual oil is retained in the columnar pore, and the capillary forces and displacement balance are reached, and the crude oil no longer changes. The dimensionless pressure difference at points P_1_ and P_2_ with displacement time is shown in Figure 17.

By combining Figure 16 and Figure 17, we can observe that at the beginning of the displacement, the displacement phase does not flow into the pore, and the dimensionless pressure difference is about 0. When the dimensionless time is 0.09, the polymer solution flows into the pore from the *f*_1_ direction, the pressure difference has a little increase, and increases to 0.09. When the dimensionless time is 0.109, the polymer solution flows through points P_1_ and P_2_, resulting in a jump in the pressure difference between the two points due to the narrow pore space and high pressure near point P_1_. When the dimensionless time is 0.113, the pores in the direction of *f*_4_ near point P_2_ are small, resulting in a sharp drop in the pressure difference between the two points. When the time is more than 0.12, the pressure difference is stable, and the column residual oil does not move.

This study found that permeability is not the only factor to determine front breakthrough; the main flow path and sorting of solid particles are other factors to determine front breakthrough in two layers of homogeneous reservoirs. Due to gravity, more residual oil can be formed in the lower layer of the vertical heterogeneous reservoir. The conditions of this study meet those of actual reservoirs; thus, these findings are significant to further improve oil recovery.

## 4. Conclusions

In this study, the micro-flow mechanism of polymer flooding has been revealed by numerical simulations, considering the shape of rock particles, wettability, and gravity. The microscopic dual heterogeneous pore model of the different rock particles was constructed. The relationship between rock particle shape on the microscopic oil displacement efficiency was characterized. The effects of different wettability conditions and the degree of wettability on the microscopic oil displacement efficiency were discussed and validated by numerical simulations. Furthermore, the formation process and conditions of columnar residual oil were described by numerical simulation results. The simulation results showed that the circular particle model with high roundness and smooth angles had the highest oil displacement efficiency at 91.57% due to the high sorting property and low flow resistance, which were 3.34% and 11.48% higher than those in the hexagonal and diamond particle models, respectively. Moreover, the circular particle with the highest oil displacement efficiency was selected to study the effects of wettability and gravity on the micro-flow mechanism. The oil displacement efficiency under water-wet conditions was greater than under oil-wet conditions, where the contact angle was closer to 90°. When gravity was considered, the oil displacement efficiency was 9.87% lower than that of no-gravity displacement. The vertical gradient caused the crude oil to crossflow to the low-permeability layer. The capillary force under water-wet conditions was found to be the driving force, and had a significant displacement effect on columnar and drop residual oil, with a higher oil displacement efficiency, even higher than 3.9% for the oil displacement efficiency under oil-wet conditions.

## Data Availability

The data presented in this study are available upon request from the corresponding author.

## Nomenclature

*Ca*	Capillary force
CT scanning technology	Computed tomography scanning technology
*Fr*	Froude number
*g*	Dimensionless gravitational acceleration
*K*	Permeability of reservoirs
*L*	Characteristic length, m
*n*	Interface normal vector
*n*_w_	Wall unit normal vector
*p*	Dimensionless pressure
*Re*	Reynolds number
*t*_w_	Wall unit tangent vector
***U***	Dimensionless velocity vector
*V*	Characteristic velocity, m/s
*We*	Weber number
*α*	Phase fraction of two phases
***κ***	Mean curvature of free surface
*μ*	Dynamic viscosity of fluid, Pa·s
*μ*_1_	Dynamic viscosity of displacement fluid, Pa·s
*μ*_2_	Dynamic viscosity of displaced fluid, Pa·s
*θ*	Contact angle
*ρ*	Density of fluid, kg/m^3^
*ρ*_1_	Density of displacement fluid, kg/m^3^
*ρ*_2_	Density of displaced fluid, kg/m^3^
*σ*	Interfacial tension, N/m
